# Percutaneous Closure of a Ruptured Sinus of Valsalva Aneurysm to Right Atrium Fistula

**DOI:** 10.1155/cric/6634261

**Published:** 2025-09-25

**Authors:** Nicholas P. Kondoleon, Andres Palomo, Justin Armstrong, Elvis Cami, Ivan D. Hanson

**Affiliations:** Department of Cardiovascular Medicine, Corewell Health William Beaumont University Hospital, Royal Oak, Michigan, USA

**Keywords:** aortic dissection, ASD occluder, sinus of Valsalva aneurysm rupture

## Abstract

Ruptured sinus of Valsalva aneurysm is a life-threatening problem that usually requires emergent surgical management. This case illustrates a transcatheter repair of a ruptured sinus of Valsalva aneurysm creating a fistula to the right atrium in a patient who was not a candidate for surgery.

**Summary**

• To recognize percutaneous closure of a ruptured sinus of Valsalva aneurysm (SOVA) as an alternative treatment strategy to surgical correction, understanding that complete defect closure may not be possible given the limitations of currently available devices.
• To understand the technical aspects involved when utilizing an Amplatzer occluder device to percutaneously close a ruptured SOVA.

## 1. History of Presentation

A 76-year-old man presented to the ED with sharp chest pain radiating to the back. Physical examination revealed tachycardia, regular rhythm, clear lung fields to auscultation, and no peripheral edema. JVP was elevated to > 20 cm, and a pancardiac murmur was noted, loudest at the left lower sternal border. He subsequently developed hypotension, obtundation, and decreased urine output, consistent with cardiogenic shock.

## 2. Past Medical History

The patient's history included a recently diagnosed 6.0 cm SOVA without follow-up; untreated multiple myeloma; surgical aortic valve replacement with bioprosthetic valve; however, this was completed out of state with no information on the reason for replacement or surgical details; coronary artery bypass graft (CABG) with LIMA-LAD and SVG-diagonal; ischemic cardiomyopathy; paroxysmal atrial fibrillation with pulmonary vein isolation and surgical left atrial appendage closure; CKD III; and hypertension.

## 3. Differential Diagnosis

The leading diagnosis was aortic dissection. However, acute coronary syndrome could not be ruled out in the setting of chest pain, coronary artery disease (CAD), and cardiogenic shock.

## 4. Investigations

**Figure 1:**
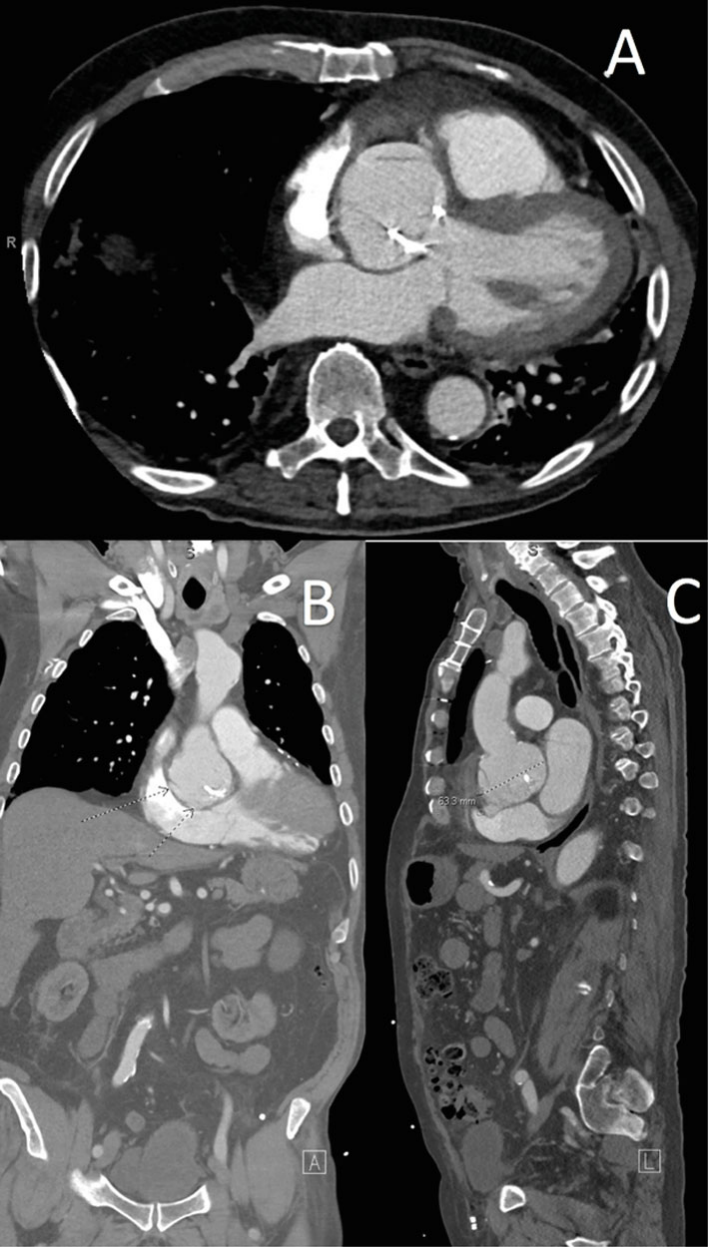
(A–C) Computed tomography angiography of the chest. Demonstration of a 6.7-cm aneurysmal dilation of the aortic root with a thin dissection and active contrast extravasation into the right atrium.

**Figure 2:**
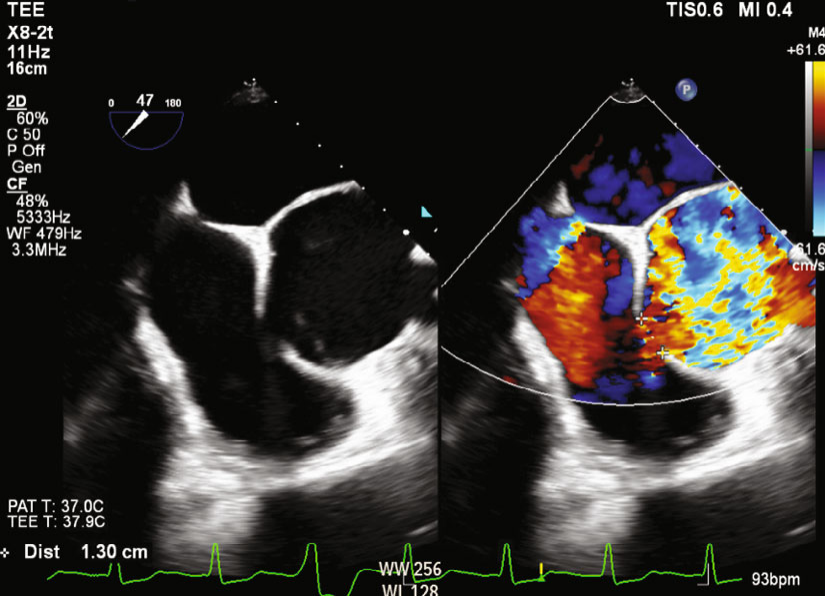
Diagnostic transesophageal echocardiogram. Demonstration of a 6.5-cm aortic root with a dissection flap into the wall of the right coronary cusp and rupture into the right atrium.

CTA confirmed a 6.7-cm aortic root with a dissection flap extending along the right sinus of Valsalva. There was contrast extending inferiorly with active extravasation into the right atrium and an aneurysmal neck diameter of 17 mm (Figure 1). Evaluation of the coronary arteries by CTA showed severe native vessel disease with a patent LIMA graft and a SVG-diagonal graft. There was no involvement of the dissection into the RCA. The electrocardiogram was normal. Transthoracic echocardiography (TTE) demonstrated a severely enlarged aortic root with rupture of the right coronary cusp and continuous flow into the right atrium. The right ventricle was moderately enlarged with mildly decreased systolic function. Left ventricular ejection fraction was 55% with hypokinesis of the mid inferior and inferolateral walls. Transesophageal echocardiogram (TEE) revealed dilated right and left atria, a small secundum ASD with bidirectional flow, and a dissection flap into the wall of the right coronary cusp and rupture into the right atrium. The defect on the atrial side measured 0.7 × 0.6 cm and demonstrated systolic and diastolic flow (Figure 2). There were no clinical signs of infection thought to be contributing to the patient's clinical presentation.

## 5. Management

The patient was not a candidate for surgical repair due to age, redo status, CKD, and untreated multiple myeloma. As a result, the decision was made to attempt transcatheter repair.

Right common femoral vein access was obtained with a 7F sheath, and right and left femoral arteries with a 6F sheath. Initial right heart catheterization revealed right atrial (RA) pressure of 10 mmHg, right ventricular (RV) pressure of 50/9 mmHg, pulmonary artery (PA) pressure of 48/15 mmHg (28), PA saturation of 81.8%, and wedge (PCWP) of 8 mmHg. Fick CO/CI (Qs): 6.2 L/min; 2.9 L/min/m^2^. Fick CO/CI (Qp): 11.4 L/min; 5.38 L/min/m^2^. Qp : Qs = 1.8.

The fistula was crossed with a multipurpose diagnostic catheter and a 0.035“J wire.” The J wire was then exchanged for a 0.035 Supercore wire and positioned in the right atrium.

Via the right femoral venous sheath, a JR4 guide catheter was advanced into the right atrium. An Ensnare was then used to snare the Supercore wire and externalize it from the right femoral vein (Supporting Information 1: Video S1).

The 7F sheath in the right femoral vein was replaced with an 18F DrySeal sheath. A 9F TorqVue sheath was advanced across the defect from the venous side, and a 20 mm Amplatzer Septal Occluder was deployed uneventfully (Supporting Information 2: Video S2 and Supporting Information 3: Video S3). When choosing a device for the fistula, an Amplatzer atrial septal occluder was chosen. With a 20-mm waist diameter (slightly larger than the 17 mm defect size measured on CTA), a 34-mm aortic disc, and a 30-mm RA disc, this device was believed to fill the defect adequately while providing large enough retention discs to prevent embolization. There was residual shunting around the RA disc when the delivery cable was still attached, but there was negligible color flow around the device after it was released.

A postdevice right heart catheterization revealed RA 8 mmHg, RV 41/8 mmHg, PA 74.4%, Fick CO/CI: 7.7 L/min:3.75 L/min/m^2^, and Qp : Qs = 1.0. The Baim Turi would not advance easily into the PA, and no wedge was obtained.

In the days following percutaneous closure, the patient developed dyspnea, hemolytic anemia, and signs of right-sided heart failure. A repeat right heart catheterization revealed RA 12 mmHg, RV 45/5 mmHg, PA 48/20 mmHg (mean 33), PCWP 15 mmHg, and Qp:Qs 1.65.

Repeat TEE revealed his known small secundum ASD with trace flow and a well-seated occluder device with a moderately sized peridevice leak (Supporting Information 4: Video S4, Supporting Information 5: Video S5, and Supporting Information 6: Video S6). His clinical state was attributed to residual shunting with inadequate diuresis. The patient was treated with intravenous iron, pentoxifylline, clopidogrel, and furosemide. He was discharged to an acute rehab facility on Oostop Day 24.

## 6. Discussion

SOVA is rare, comprising 0.09% of the general population, with 0.1%–3.5% due to congenital etiologies [1]. Unruptured aneurysms are generally asymptomatic; however, symptoms can manifest due to compression of local structures, such as coronary arteries and the conduction system [2]. Rupture of SOVA can occur in 35% of patients and can present with chest pain, shortness of breath, and fatigue, determined by the size of the shunt, presence of associated lesions, and age [3]. If left untreated, patients with ruptured SOVA have a median survival rate of 2 years, succumbing to their disease state due to heart failure and left to right shunting [4, 5].

There are no established guidelines for treatment of SOVA; however, it is generally accepted to follow aortic root aneurysm guidelines. According to the 2022 AHA guidelines on management of aortopathies, the recommended approach to management of ruptured thoracic aortic aneurysms is surgical repair; however, endovascular stenting may be reasonable in hemodynamically stable patients with appropriate anatomy [6]. Currently, patients with no previous cardiothoracic surgical history carry a mortality rate of 1.9%–3.6% with a 90% survival rate at 15 years [1].

Over the past two decades, reports have emerged describing percutaneous closure strategies as an attractive alternative to managing ruptured SOVA. Sinha et al. describe eight patients with an 87.5% closure rate. At 55 months follow-up, there was no residual shunt, progression of aortic regurgitation (AR), new AR, infective endocarditis, and device embolization, and all patients were NYHA I [7]. Galeczka et al. reported procedural success in 19/23 patients with improvement in NYHA class symptoms in all but two patients, and three patients required further percutaneous intervention for residual shunting and recurrent ruptured SOVA. No mortality was noted in follow-up [8]. Finally, Kerkar et al. treated 20 patients with a 90% procedural success rate. Five patients had residual shunting, and four patients had trivial AR. At 24 months follow-up, all patients were NYHA I–II, residual shunting resolved in 3/5, procedure-related AR resolved in two of four patients, and there was no AR progression, endocarditis, device embolization, or mortality [9].

Hemolysis from intracardiac prosthetic devices requiring blood transfusions occurs in 1%–2% of cases [10]. Medical therapy is appropriate in mild hemolysis; however, invasive management may be required for severe symptomatic hemolysis. In a small randomized trial including 40 patients, the use of pentoxifylline improved hemolysis indices by 60% compared to 5% in the placebo group [10]. In this case, conservative medical management of hemolysis was pursued due to the risk of dislodging the recently deployed closure device with repeat transcatheter closure attempts. Even wire crossing of the residual defect was felt to be prohibitive due to the uncertain course of the serpiginous leak.

## 7. Follow-Up

Two weeks following discharge, the patient had improving anemia, stabilization of kidney function, resolution of dyspnea, and improvement in functional capabilities.

## 8. Conclusions

Percutaneous closure of a ruptured SOVA with fistula into the right atrium is feasible, although there are no transcatheter devices that are purpose-built for this type of defect. As such, incomplete closure may be the best possible outcome, but it may still increase the likelihood of survival in the short term. Pentoxifylline may be useful for device-related hemolysis that may be associated with incomplete defect closure [11].

## Data Availability

Data sharing is not applicable to this article as no new data were created or analyzed in this study.

## Nomenclature

SOVA: sinus of Valsalva aneurysm
SCAI: Society for Cardiovascular Angiography and Interventions
CTA: computed tomography angiography
CABG: coronary artery bypass graft
LIMA: left internal mammary artery
LAD: left anterior descending
SVG: saphenous vein graft
LCx: left circumflex
RCA: right coronary artery
Qp:Qs: pulmonary:systemic flow
LVOT: left ventricular outflow tract

## Supporting Information

Supporting Information 1 Video S1: Crossing of fistula and externalization of wire. The wire was advanced across the fistula via femoral artery, into the aorta, through the fistula, and into the right atrium. The wire was then snared in the right atrium and externalized.

Supporting Information 2 Video S2: Positioning of the atrial septal occluder device. Over the externalized wire, a sheath was advanced across the fistula from the femoral vein, through the fistula, and into the aorta. The atrial septal occluder was then advanced through the sheath, exposing the distal retention disk. The sheath and occluder were retracted en bloc, such that the distal retention disk engaged the luminal surface of the aorta. At that point, the proximal retention disk was exposed, “sandwiching” the aortic wall and free wall of the right atrium together and closing the defect.

Supporting Information 3 Video S3: Fully deployed atrial septal occluder device. The atrial septal occluder is fully deployed and released from the delivery system.

Supporting Information 4 Video S4: Postop transesophageal echocardiogram. Modified mid-esophageal aortic valve short axis view demonstrating a well-seated occluder device, at the level of the sinus of Valsalva into the right atrium, with a moderately sized peridevice leak.

Supporting Information 5 Video S5: Postop transesophageal echocardiogram. Modified transgastric view demonstrating a well-seated occluder device at the level of the sinus of Valsalva to the right atrium, significantly occluding the previously seen fistula.

Supporting Information 6 Video S6: Postop transesophageal echocardiogram. Modified transgastric view with color Doppler demonstrating a well-seated occluder device at the level of the sinus of Valsalva to the right atrium with a moderately sized peridevice leak.
